# The Prevalence of Ureaplasma Urealyticum and Mycoplasma Hominis Infections in Infertile Patients in the Northeast Region of Romania

**DOI:** 10.3390/medicina57030211

**Published:** 2021-02-26

**Authors:** Bogdan Doroftei, Ovidiu-Dumitru Ilie, Theodora Armeanu, Emil Anton, Ioana Scripcariu, Radu Maftei

**Affiliations:** 1 Faculty of Medicine, University of Medicine and Pharmacy “Grigore T. Popa”, University Street, no 16, 700115 Iasi, Romania; bogdandoroftei@gmail.com (B.D.); theodoraarmeanu@yahoo.com (T.A.); emil.anton@yahoo.com (E.A.); isscripcariu@gmail.com (I.S.); dr.radu.maftei@gmail.com (R.M.); 2 Clinical Hospital of Obstetrics and Gynecology “Cuza Voda”, Cuza Voda Street, no 34, 700038 Iasi, Romania; 3 Origyn Fertility Center, Palace Street, no 3C, 700032 Iasi, Romania; 4 Department of Biology, Faculty of Biology, “Alexandru Ioan Cuza” University, Carol I Avenue, no 20A, 700505 Iasi, Romania

**Keywords:** Ureaplasma urealyticum, Mycoplasma hominis, prevalence, drug susceptibility, infertility

## Abstract

*Background and objectives:* Ureaplasma urealyticum (UU) and Mycoplasma hominis (MH) are two commensal microorganisms that form the urogenital microbiota. Under a state of dysbiosis, both bacteria cause intrauterine infection. *Material and methods:* Therefore, the purpose of the present study was to analyze the prevalence of UU and MH among four hundred and eleven infertile women. *Results:* Women between thirty and thirty-five years old were the most affected group, followed by those that were 25 and 30 years old, respectively. Cumulatively, the prevalence of single UU and MH, and coinfection, was 28.46% (*n* = 117), (*n* = 2) 0.48%, and 2.91% (*n* = 12), respectively, with an overall detection rate of 31.87% (*n* = 131). To assess the associated drug susceptibility, endocervical samples were unequally sent to Regina Maria (*n* = 281) and Synevo (*n* = 130) laboratories for further analyses. Pristinamycin (100% vs. 100%) and Josamycin (100% vs. 98.00%) were the most efficient antibiotics in eradicating UU and MH, several others also displaying a high efficiency, among which can be mentioned Doxycycline (98.23%), Minocycline (96.00%), Tetracycline (96.48% vs. 68.00%), and Erythromycin (70.17% vs. 92.00%). Based on antibiograms, Clarithromycin (88.00%), Roxithromycin (88.00%), Levofloxacin (82.00%), and Azithromycin (78.94%) can be further used in treating such infections. On the other hand, Clindamycin (4.00%) and Ciprofloxacin (12.27% vs. 2.00%) are no longer viable because both UU and MH display an intermediate response towards gained resistance. Interestingly, the efficiency of Ofloxacin (22.79% vs. 60.00%) was conflicting, this possibly suggesting a transient stage to a gradual adaptability of these microorganisms to Ofloxacin. *Conclusions:* The most susceptible age groups in each case were women that were between twenty and forty years old. It can be concluded that four antibiotics can be safely used for treating UU, MH, or dual infections whose efficiency was over 95%.

## 1. Introduction

In conjunction with the World Health Organization’s (WHO) directives, infertility represents the incapacity to conceive a baby after one year of unprotected sexual activity before the age of thirty-five. However, there are disagreements in terms of definitions since WHO suggests at least two years of intercourse to validate these guidelines [1].

Infertility is an increasingly expanding topic of interest that brought solid information regarding its origin. It was concluded through the prism of all available evidence that infertility possesses a multifactorial substrate, reflected by an impaired physiologic environment [2].

The latest figures issued indicate that 10%–20% of cases are attributed to male infertility, 30%–40% for both sexes [3], and infections at the genital tract are responsible for 15% of cases in men [4]. Therefore, it should not be omitted that the incidence of sexually transmitted diseases (STDs) has risen in recent decade(s). Left untreated, the consequences are characterized by a series of other, potentially life-threatening pathologies [5,6,7].

Here, we refer to Mycoplasma hominis (MH) and Ureaplasma urealyticum (UU), whose high tropism is positively associated with various disorders [8,9] even in asymptomatic patients [10,11], some of them being irreversible. Stellrecht et al. [12] revealed in 2004 that 40% of infants born from carrier mothers of MH and UU were diagnosed with neonatal conjunctivitis and meningitis. The authors maintain that an ascending route crossing the placenta via the birth canal could exist.

Even though in the current literature, a substantial number of reports regarding the worldwide incidence of genital mycoplasmas infections can be found, studies that aim to establish the prevalence, antibiotic resistance patterns, types, and transmission of MH and UU in Romania are limited or do not exist [13].

Such a study is critical, especially since Romania ranks first in Europe in terms of adolescent pregnancy. Thus, the study aims to determine the prevalence of MH and UU infections among infertile patients in the northeast region of Romania. Considering the potential of microorganisms to gain resistance against antibiotics, we also want to contour the antimicrobial susceptibility to a series of antagonists for these two microorganisms.

## 2. Materials and Methods

### 2.1. Study Participants

This study was conducted between 2017 and 2019 at the Origyn Fertility Center in Iasi, Romania, an interval during which a total of four hundred and eleven women were included (mean 31.85, range 18–51 years old). The main inclusion criteria were: (I) a confirmed diagnosis of infertility, and (II) absence of Chlamydia trachomatis infection. There was no maximum age limit for participation in this study.

### 2.2. Sample Collection and Processing

All endocervical samples were collected from patients using an Aptima Unisex Swab Specimen Collection Kit for Endocervical and Male Urethral Swab Specimens (San Diego, CA 92121, USA). As we continued with the investigation, samples were transferred to Synevo (*n* = 130) and Regina Maria (*n* = 281) laboratories for further analysis. Synevo laboratory used Mycoplasma IES (Autobio Diagnostics, Zhengzhou, China) to identify and test the antimicrobial susceptibility of UU and MH to 11 antibiotics, namely, Macrolide class (Clarithromycin, Erythromycin, Joysamicin, Roxithromycin), Fluoroquinolones (Ciprofloxacin, Ofloxacin, Levofloxacin), Tetracycline (Minocycline, Tetracycline), Streptogramins (Pristinamycin), and Lincosamides (Clindamycin). Regina Maria laboratory used Mycoplasma IST2 (bioMérieux, Marcy-l’Etoile, France) to detect and assess the sensitivity of MH and UU to 8 antibiotics, members of 4 district classes, namely, Macrolide class (Josamycin, Erythromycin, Azithromycin), Tetracycline (Doxycycline, Tetracycline), Fluoroquinolones (Ofloxacin, Ciprofloxacin), and Streptogramins (Pristinamycin).

### 2.3. Ethical Approval

The present study was approved by the local committee of the Orygin Fertility Center, Iasi, Romania (no 115/565; 25 January 2021). This manuscript was conducted in accordance with the Helsinki Declaration of human rights and national and European regulations on biomedical research in force. Participants were kindly asked and agreed to sign written informed consent forms for their voluntary contribution to this study. They did not receive any remuneration in this context.

### 2.4. Statistical Analysis

Data analysis was carried out using Microsoft Excel 2010 and Minitab 19 software (Minitab Inc., State College, Pennsylvania, USA, 2019). Microsoft Excel was used for editing, sorting, and coding. Statistical analysis was performed with Minitab 19.

## 3. Results

**Figure 1:**
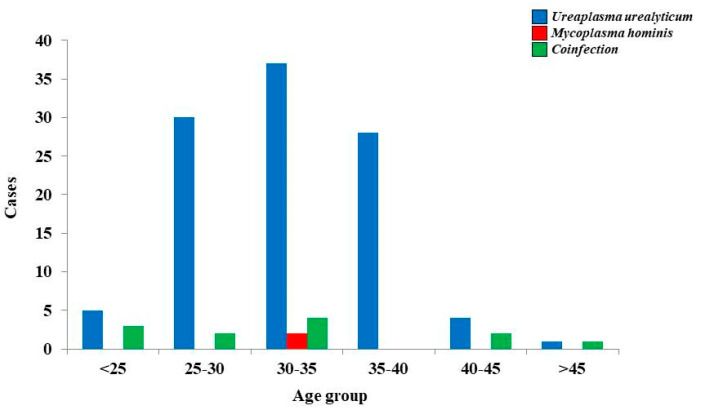
Prevalence of Ureaplasma urealyticum and Mycoplasma hominis infection by age.

The prevalence of MH and UU was (*n* = 2) 0.48% and 28.46% (*n* = 117). We also noted that 2.91% (*n* = 12) of all participants had coinfections. The overall detection rate was up to 31.87% (*n* = 131). There is a fluctuating tendency of UU infections among early adulthood and middle-aged women. We identified only two cases characterized strictly by an MH infection within the group between 30 and 35 years old. Even though coinfections were reported in a small percentage if we refer to associated figures for UU, the pattern in terms of predisposition is identical to that of UU affecting the same age groups (Figure 1).

**Figure 2:**
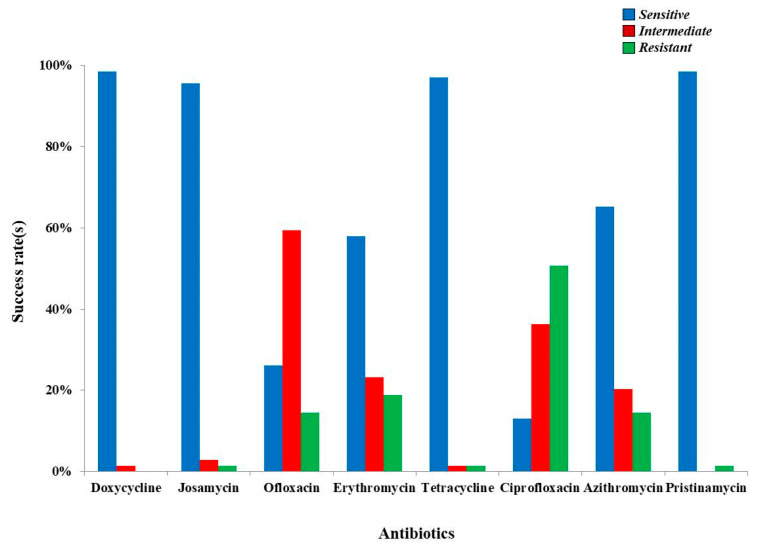
Drug susceptibility of Ureaplasma urealyticum and Mycoplasma hominis based on antibiograms from Regina Maria.

Samples were unequally sent to other institutions for additional analyses. From the total number of samples collected, 68.46% (*n* = 281) were analyzed within Regina Maria laboratory. The overall detection rate was 20.28% (*n* = 57), data detailed in Table 1. Pristinamycin and Josamycin possessed full efficiency (100%) against UU and MH. Furthermore, Doxycycline (98.23%) and Tetracycline (96.48%) are highly effective and can be used as agents dedicated to eradicating the presence of these pathogens. Azithromycin and Erythromycin also hold a potent reactivity rate that reached 78.94% and 70.17%. In contrast with the above-mentioned, Ciprofloxacin and Ofloxacin can no longer be considered suitable, since their potency is variable, reflected by an intermediate response (61.40%) towards the gained resistance status (50.87%) of UU. (Figure 2). The most susceptible groups were women that were 20 and 40 years old, figures that clearly highlight a direct correlation between the presence of UU and MH and age (Table 1).

**Figure 3:**
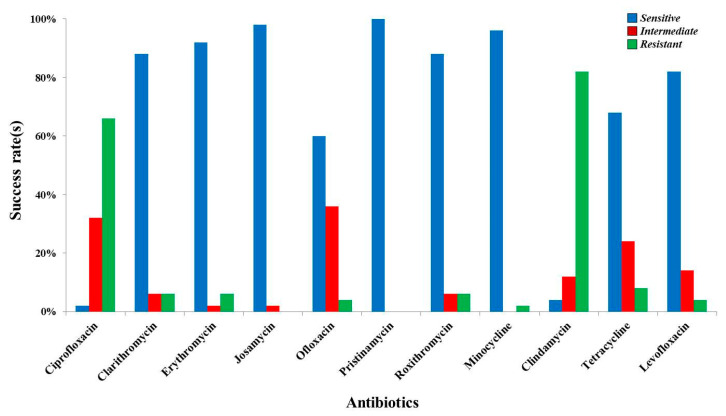
Drug susceptibility of Ureaplasma urealyticum and Mycoplasma hominis based on antibiograms from Synevo.

Synevo laboratory was responsible for processing 31.54% of the samples (*n* = 130), the number of cases in which we had cogent data being only fifty. Despite the wide spectrum of antibiotics used, Pristinamycin was the sole antibiotic with complete efficiency. Except for Erythromycin and Josamycin, with viability over 90%, Minocycline had a 96% success rate against UU. Clarithromycin and Roxithromycin exerted beneficial activity (88.00%) in countering the persistence of UU, further highlighting a high sensitivity of this microorganism to Levofloxacin (82.00%). As indicated in Table 2, the susceptibility of UU to Tetracycline was 68.00%, significantly lower if we refer to the results reported previously. The data are contradictory for Ofloxacin (60.00%), since above it had only 19.29% sensitivity. Unfortunately, Clindamycin and Ciprofloxacin were ineffective in 66.00% and 82.00% of cases for infections with UU (Figure 3). Analogous to our previous results, the most susceptible groups were also women that were 20 to 40 years old, a downward trend being positively correlated with age (Table 2).

## 4. Discussion

As presented throughout this manuscript, Ureaplasma urealyticum was prevalent among all samples analyzed. The single UU, MH, and dual infections accounted for 28.46% (*n* = 117), (*n* = 2) 0.48%, and 2.91% (*n* = 12), respectively. Even though Ureaplasma urealyticum and Mycoplasma hominis are non-pathogenic commensal bacteria, part of the normal genital flora, some factors could perturb the host’s eubiosis [14].

These arguments were strengthened on two distinct occasions. UU was identified in ≥1 site in 52.9% of the cases, while MH in 3.3%, but always in association with UU. Figures reached 23% in cord blood cultures, being more common in infants of nonwhite women under twenty years of age (27.9% vs. 16.8%; *p* = 0.016). Even though the cultures were initially positive in cases of spontaneous, preterm, or early gestation age (34.7% vs. 3.2%; *p* = 0.001), the therapeutic regimen led to a depletion of these strains [15]. This topic should be of interest because elevated levels of interleukin-6 and placental histology were noted, showing that the risk of systemic inflammation (41.3% vs. 25.7%; *p* = 0.007), funisitis [16], and bronchopulmonary dysplasia (26.8% vs. 10.1%; *p* = 0.0001) is significantly higher in neonates [17].

In order to offer a conclusive overview regarding all studies performed to date with a similar objective to our manuscript, we performed some searches in available databases. We identified five studies in which the teams of authors included a total number of 34,698 Chinese participants. Cumulatively, the scholars demonstrated that UU had a prevalence of 22.01% (*n* = 7639), 2.37% MH (*n* = 824), and 3.74% coinfection (*n* = 1300). One common observation was that women are more predisposed than men (*p* < 0.001), regardless of age and health status, also inflicting changes in semen parameters. Josamycin, Clarithromycin, Roxithromycin, Doxycycline, Minocycline, and Tetracycline could be recommended in both symptomatic and asymptomatic individuals for clinically treating UU and MH infections since the success rate of these three antibiotics was 94.6%, 100%, and 84.3%, respectively [18,19,20,21,22].

Concerning sperm integrity, Lee et al. [23] were the first that proved the existence of a positive correlation between UU and sperm integrity. More precisely, UU was prevalent among infertile individuals of both sexes (*p* = 0.022), 48% from infertile men and 40% in infertile women, whereas the figures were 25% and 22.9% in fertile men and women, respectively. Related conclusions were expressed by Salmeri et al. [24], since 41% infertile men that presented to a clinic had urogenital symptoms (UU—*n* = 39; MH—*n* = 9; mixed—*n* = 1), oligo-astheno-teratozoospermia (Chi-square = 127.3; *p* < 0.05), and asthenozoospermia (Chi-square = 5.74; *p* < 0.05), in contrast to non-infected infertile patients.

Moridi et al. [25] conducted a systematic review and meta-analysis in 2019 regarding the prevalence of UU and MH in Iran in the last 19 years. Based on a total of forty-four articles selected for data extraction, the authors concluded that UU and MH prevalence was 17.53% and 9.68%, respectively, especially in central provinces compared to in other parts of Iran.

Even though antibiotics proved their efficiency, the beneficial activity of lactic bacteria remained under-explored, yet crucial, having major clinical implications. Daniele et al. [26] concluded that bacteriocins L23 and L60 produced by Lactobacillus fermentum and Lactobacillus rhamnosus are two novel inhibitors of bacterial infection. The minimum inhibitory concentration (MIC) of L23 ranged between 320 and 160/80 UA mL^−1^ for 78%/95% of the MH and UU, respectively, whereas L60 was still active at 160/80 UA mL^−1^ for 56%/53% of the MH and UU infection, respectively.

Fortunately, we possess the knowledge and equipment to detect both UU and MH from a molecular point of view, polymerase chain reaction (PCR) remaining the method of choice due to its high specificity and sensitivity as opposed to direct fluorescence antibody (DFA) [27,28,29].

It is worth mentioning that interstitial cystitis/painful bladder syndrome (IC/PBS) is associated with endometriosis and infertility. According to a recent review by Garzon et al. [30], there is no reliable strategy for IC/BPS due to scarce evidence and limited approaches. Unfortunately, current methodologies are not applied in a personalized manner and depend on pathophysiological causes. The only applied technique involves the use of conservative options that are more invasive, targeting the bladder.

Patnaik et al. [31] conducted another review referring to the underlying mechanisms of IC/PBS. Congruent with the conclusions reached by Garzon and co-authors, the etiology is not clearly understood. They estimated the prevalence to be in the range of 45/8 per 100,000 in women and men. The joint prevalence in both sexes was up to 10.6 cases per 100,000. A series of etiological theories were issued to find novel diagnostic strategies and biomarkers. They concluded that there are no “gold standards” of IC/PBS, the only alternative in the future remaining to classify the patients based on symptoms and with emphasis on the phenotype.

## 5. Conclusions

It can be concluded that the persistence of Mycoplasma hominis and Ureaplasma urealyticum exerts a detrimental activity in certain circumstances, such as dysbacteriosis or other pathologies, by negatively affecting the human fertility status. We can successfully reduce infections and infertility risks by educating and screening the population, and adequately treating individuals who test positive. Avoiding unreliable treatment of these infections contributes to reducing multidrug resistance and conserving drug susceptibility.

## Figures and Tables

**Table 1:** Data revealing the susceptibility of Ureaplasma urealyticum and Mycoplasma hominis depending on s = sensitivity, i = intermediate, and r = resistant status (Regina Maria).

Antibiotics	UU (Positive, *n* = 55) and MH (Positive, *n* = 2)					
	Cumulative Results		Results based on the Age			
			20–30 (*n* = 19)	30–40 (*n* = 33)	40–50 (*n* = 4)	>51 (*n* = 1)
**Doxycycline**	S = 54; 94.73%	S = 2; 3.50%	S = 18	S = 33	S = 4	S = 1
	I = 1; 1.75%	I = 0; 0.00%	I = 1	I = 0	I = 0	I = 0
	R = 0; 0.00%	R = 0; 0.00%	R = 0	R = 0	R = 0	R = 0
**Josamycin**	S =55; 96.49%	S = 2; 3.50%	S= 19	S = 33	S = 4	S = 1
	I = 0; 0.00%	I = 0; 0.00%	I = 0	I = 0	I = 0	I = 0
	R = 0; 0.00%	R = 0; 0.00%	R = 0	R = 0	R = 0	R = 0
**Ofloxacin**	S = 11; 19.29%	S = 2; 3.50%	S = 4	S = 9	S = 0	S = 0
	I = 35; 61.40%	I = 0; 0.00%	I = 12	I = 19	I = 3	I = 1
	R = 9; 15.78%S	R= 0; 0.00%	R = 3	R = 5	R = 1	R = 0
**Erythromycin**	S = 40; 70.17%	S = 0; 0.00%	S = 13	S = 24	S = 2	S = 1
	I = 14; 24.56%	I = 0; 0.00%	I = 6	I = 6	I = 2	I = 0
	R = 1; 1.75%	R = 2; 3.50%	R = 0	R = 3	R = 0	R = 0
**Tetracycline**	S = 53; 92.98%	S = 2; 3.50%	S = 17	S = 33	S = 4	S = 1
	I = 1; 1.75%	I = 0; 0.00%	I = 1	I = 0	R = 0	I = 0
	R = 1; 1.75%	R = 0; 0.00%	R = 1	R = 0	I = 0	R = 0
**Ciprofloxacin**	S = 5; 8.77%	S = 2; 3.50%	S = 1	S = 6	S = 0	S = 0
	I = 21; 31.84%	I = 0; 0.00%	I = 7	I = 13	I = 0	I = 1
	R = 29; 50.87%	R = 0; 0.00%	R = 11	R = 14	R = 4	R = 0
**Azithromycin**	S = 45; 78.94%	S = 0; 0.00%	S = 17	S = 25	S = 2	S = 1
	I = 10; 17.54%	I = 0; 0.00%	I = 2	I = 6	I = 2	I = 0
	R = 0; 0.00%	R = 2; 3.50%	R = 0	R = 2	R = 0	R = 0
**Pristinamycin**	S = 55; 96.49%	S = 2; 3.50%	S = 19	S = 33	S = 4	S = 1
	I = 0; 0.00%	I = 0; 0.00%	I = 0	I = 0	I = 0	I = 0
	R = 0; 0.00%	R = 0; 0.00%	R = 0	R = 0	R = 0	R = 0
			**Coinfection (*n* = 12)**			
			**20–30** **(*n* = 5)**	**30–40** **(*n* = 4)**	**40–50** **(*n* = 3)**	**>51** **(*n* = 0)**
			S = 5	S = 4	S = 3	S = 0
			I = 0	I = 0	I = 0	I = 0
			R = 0	R = 0	R = 0	R = 0
			S = 4	S = 3	S = 2	S = 0
			I = 1	I = 1	I = 0	I = 0
			R = 0	R = 0	R = 1	R = 0
			S = 2	S = 2	S = 1	S = 0
			I = 3	I = 1	I = 2	I = 0
			R = 0	R = 1	R = 0	R = 0
			S = 0	S = 0	S = 0	S = 0
			I = 0	I = 1	I = 1	I = 0
			R = 5	R = 3	R = 2	R = 0
			S = 5	S = 4	S = 3	S = 0
			I = 0	I = 0	I = 0	I = 0
			R = 0	R = 0	R = 0	R = 0
			S = 0	S = 1	S = 1	S = 0
			I = 3	I = 1	I = 0	I = 0
			R = 2	R = 2	R = 2	R = 0
			S = 0	S = 0	S = 0	S = 0
			I = 2	I = 1	I = 1	I = 0
			R = 3	R = 3	R = 2	R = 0
			S = 5	S = 4	S = 3	S = 0
			I = 0	I = 0	I = 0	I = 0
			R = 0	R = 0	R = 0	R = 0

**Table 2:** Data revealing the susceptibility of Ureaplasma urealyticum depending on s = sensitivity, i = intermediate, and r = resistant status (Synevo).

Antibiotics	UU (Positive, *n* = 50) and MH (Positive, *n* = 0)					
	Cumulative Results		Results based on the Age			
			20–30 (*n* = 16)	30–40 (*n* = 33)	40–50 (*n* = 1)	>51 (*n* = 0)
**Ciprofloxacin**	S = 1; 2.00%	S = 0; 0.00%	S = 0	S = 1	S = 0	S = 0
	I = 16; 32.00%	I = 0; 0.00%	I = 6	I = 10	I = 0	I = 0
	R = 33; 66.00%	R = 0; 0.00%	R = 10	R = 22	R = 1	R = 0
**Clarithromycin**	S = 44; 88.00%	S = 0; 0.00%	S =14	S = 29	S = 1	S = 0
	I = 3; 6.00%	I = 0; 0.00%	I = 1	I = 2	I = 0	I = 0
	R = 3; 6.00%	R = 0; 0.00%	R = 1	R = 2	R = 0	R = 0
**Erythromycin**	S = 46; 92.00%	S = 0; 0.00%	S = 15	S = 30	S = 1	S = 0
	I = 1; 2.00%	I = 0; 0.00%	I = 0	I = 2	I = 0	I = 0
	R = 3; 6.00%	R = 0; 0.00%	R = 1	R = 2	R = 0	R = 0
**Josamycin**	S = 49; 98.00%	S = 0; 0.00%	S = 16	S = 32	S = 1	S = 0
	I = 1; 2.00%	I = 0; 0.00%	I = 0	I = 1	I = 0	I = 0
	R = 0; 0.00%	R = 0; 0.00%	R = 0	R = 0	R = 0	R = 0
**Ofloxacin**	S = 30; 60.00%	S = 0; 0.00%	S = 10	S = 19	S = 1	S = 0
	I = 18; 36.00%	I = 0; 0.00%	I = 6	I = 12	I = 0	I = 0
	R = 2; 4.00%	R = 0; 0.00%	R = 0	R = 2	R = 0	R = 0
**Pristinamycin**	S = 50; 100%	S = 0; 0.00%	S = 16	S = 33	S = 1	S = 0
	I = 0; 0.00%	I = 0; 0.00%	I = 0	I = 0	I = 0	I = 0
	R = 0; 0.00%	R = 0; 0.00%	R = 0	R = 0	R = 0	R = 0
**Roxithromycin**	S= 44; 88.00%	S = 0; 0.00%	S = 14	S = 29	S = 1	S = 0
	I = 3; 6.00%	I = 0; 0.00%	I = 1	I = 2	I = 0	I = 0
	R = 3; 6.00%	R = 0; 0.00%	R = 1	R = 2	R = 0	R = 0
**Minocycline**	S = 48; 96.00%	S = 0; 0.00%	S = 15	S = 32	S = 1	S = 0
	I = 0; 0.00%	I = 0; 0.00%	I = 0	I = 0	I = 0	I = 0
	R = 1; 2.00%	R = 0; 0.00%	R = 0	R = 1	R = 0	R = 0
**Clindamycin**	S = 2; 4.00%	S = 0; 0.00%	S = 1	S = 1	S = 0	S = 0
	I = 6; 12.00%	I = 0; 0.00%	I = 0	R = 6	I = 0	I = 0
	R = 41; 82.00%	R = 0; 0.00%	R = 15	R = 25	R = 1	R = 0
**Tetracycline**	S = 34; 68.00%	S = 0; 0.00%	S = 11	S = 23	S = 0	S = 0
	I = 12; 24.00%	I = 0; 0.00%	I = 4	I = 7	I = 1	I = 0
	R = 4; 8.00%	R = 0; 0.00%	R = 1	R = 3	R = 0	R = 0
**Levofloxacin**	S = 41; 82.00%	S = 0; 0.00%	S = 14	S = 26	S = 1	S = 0
	I = 7; 14.00%	I = 0; 0.00%	I = 2	I = 5	I = 0	I = 0
	R = 2; 4.00%	R = 0; 0.00%	R = 0	R = 2	R = 0	R = 0

## Data Availability

The datasets used and analyzed during the current study are available from the corresponding author upon reasonable request.
